# Bioinspired Stimulus Selection Under Multisensory Overload in Social Robots Using Reinforcement Learning

**DOI:** 10.3390/s25196152

**Published:** 2025-10-04

**Authors:** Jesús García-Martínez, Marcos Maroto-Gómez, Arecia Segura-Bencomo, Álvaro Castro-González, José Carlos Castillo

**Affiliations:** Systems Engineering and Automation Department, Universidad Carlos III de Madrid, Avenida de la Universidad, 30, Leganés, 28911 Madrid, Spain; jesusgar@ing.uc3m.es (J.G.-M.); arsegura@pa.uc3m.es (A.S.-B.); acgonzal@ing.uc3m.es (Á.C.-G.); jocastil@ing.uc3m.es (J.C.C.)

**Keywords:** bioinspired attention system, reinforcement learning, human–robot interaction, social robotics, multisensor systems, multimodal interaction

## Abstract

Autonomous social robots aim to reduce human supervision by performing various tasks. To achieve this, they are equipped with multiple perceptual channels to interpret and respond to environmental cues in real time. However, multimodal perception often leads to sensory overload, as robots may receive numerous simultaneous stimuli with varying durations or persistent activations across different sensory modalities. Sensor overstimulation and false positives can compromise a robot’s ability to prioritise relevant inputs, sometimes resulting in repeated or inaccurate behavioural responses that reduce the quality and coherence of the interaction. This paper presents a Bioinspired Attentional System that uses Reinforcement Learning to manage stimulus prioritisation in real time. The system draws inspiration from the following two neurocognitive mechanisms: Inhibition of Return, which progressively reduces the importance of previously attended stimuli that remain active over time, and Attentional Fatigue, which penalises stimuli of the same perception modality when they appear repeatedly or simultaneously. These mechanisms define the algorithm’s reward function to dynamically adjust the weights assigned to each stimulus, enabling the system to select the most relevant one at each moment. The system has been integrated into a social robot and tested in three representative case studies that show how it modulates sensory signals, reduces the impact of redundant inputs, and improves stimulus selection in overstimulating scenarios. Additionally, we compare the proposed method with a baseline where the robot executes expressions as soon as it receives them using a queue. The results show the system’s significant improvement in expression management, reducing the number of expressions in the queue and the delay in performing them.

## 1. Introduction

Social robotics is a research field focused on developing artificial agents capable of engaging with humans through social behaviours and interactions [1,2]. These agents incorporate procedures to facilitate human–robot interactions, taking advantage of multimodal interaction’s benefits [3]. Multimodal interaction allows people to communicate and exchange information with the robot using different modalities such as voice, touch, or gestures [4]. This capacity is possible due to the numerous sensors and actuators that most actual robots can manage, significantly enhancing robot capacities and naturalness [5].

One of the emerging approaches in social robotics is the design of robots that promote the development of affective bonds with users through physical interaction, known as pet robot companions. These are intended to be used mainly in therapeutic, welfare, or educational contexts, where establishing emotional engagement can enhance the effectiveness of the interaction [6]. This approach has been explored in recent studies evaluating their potential to reduce people’s loneliness and the effects of mental disorders such as depression [7,8]. In these scenarios, the physical contact between the robot and the user has demonstrated positive effects such as improved connection and bonding [9] and increased user trust in the robot [10]. However, as Nunez et al. [11] analyse in their study and our research group previously experienced in our pet robot companion [12], physical and frequent human–robot interaction causes unexpected problems in the robot’s sensory system, derived from the overstimulation of the perception channels and the unintended activation of other sensors due to the robot’s manipulation by the user. These problems affect the robot’s responses to ambient stimuli, causing repeated undesired behaviours that might negatively influence user experience.

In practical terms, decision-making and expressiveness in pet robot companions often rely directly on perceptual input, with each stimulus linked to a specific behavioural response. These actions are typically designed with a fixed duration, meaning that once triggered, they run to completion or are queued if multiple gestures are activated in succession. As a result, reactions may become delayed or appear truncated, further reducing the naturalness of the interaction. This design becomes particularly vulnerable under sensory overload; a persistent stimulus may repeatedly trigger the same gesture, leading to rigid or unrealistic reactions. Related to this issue, the robot’s behaviour could be incoherent if repeated stimuli are incorrectly managed. For example, new inputs would be ignored if the repeated stimulus arrives faster than the robot’s capacity to perform the associated reactions or queued if the system prioritises earlier ones. Similarly, when several stimuli are activated simultaneously, they compete for attention, and the robot—unable to determine priority—may execute an action misaligned with the user’s intention, resulting in inappropriate responses that undermine naturalness and, ultimately, user acceptance.

For example, if the user lifts the robot, the inertial sensors may trigger a gesture indicating that the robot acknowledges being lifted. Subsequently, if the user gives a verbal command such as asking the robot to sleep, the speech recognition module would initiate a resting behaviour. Finally, a stroke on the robot’s head could be expected to elicit a response. However, when these stimuli overlap, the robot’s behaviour can be incoherent since the robot might not know which stimulus to attend to first or which one is more relevant. This issue results in delayed and erratic responses, particularly under multisensory stimulation, and highlights the need for mechanisms that mitigate sensory overload and ensure more natural interactions.

These limitations highlight the need to determine how sensory information is prioritised in physically interactive robots. Instead of reacting equally to all perceived inputs, robots should be equipped with attentional mechanisms to regulate sensory processing based on relevance and context. We argue that addressing this challenge requires an interdisciplinary approach combining cognitive psychology and neuroscience insights to understand how humans regulate attention and manage multisensory information in real-world scenarios. Incorporating this into robotics could enable the development of systems that allow robots to prioritise relevant stimuli while filtering out redundant or persistent inputs, improving their responsiveness, naturalness, and interaction quality.

Humans are constantly surrounded by ambient stimuli, such as sounds, movements, temperature, or visual cues, that compete to grab our attention [13]. These stimuli are typically perceived simultaneously and require specialised processing to interpret and use them correctly [14]. The human brain is a powerful and specialised system that prioritises important stimuli relevant to our condition, filtering the ones that are redundant or not significant for our current state, thanks to several cognitive attentional mechanisms [13]. The capacity to manage such huge perception data allows us to efficiently maintain attentional stability, adapting ourselves to dynamic and challenging environments [15].

Building on this inspiration, the contribution of this article is the application of biologically inspired mechanisms to regulate the importance of ambient stimuli in pet-like social robots using Reinforcement Learning (RL) to reduce overstimulation and repeated sensor activation for a coherent, responsive, and real-time behaviour. The application-oriented nature of this work is addressed by the following: (i) developing a Bioinspired Attentional System (BIAS) model that dynamically prioritises sensory inputs and computes the focus of attention under hardware and computational constraints and (ii) the integration of this system in a pet robot companion to mitigate gesture repetition, queuing, and execution delays, enhancing the fluency of the robot’s responsive behaviours.

Our approach draws on two neurocognitive mechanisms—Attentional Fatigue (AF) and Inhibition of Return (IOR)—which are formally encoded into the reward function of the RL algorithm. AF refers to the gradual reduction of attention to repeated or homogeneous stimuli (e.g., filtering background noise at a party [16] or adapting to summer heat [17]), allowing cognitive resources to shift away from information that is no longer relevant [18]. IOR, in turn, decreases the salience of stimuli that remain active once they have been attended, such as becoming unaware of clothes touching the skin, favouring novel or changing inputs [19]. Integrating these mechanisms into the RL framework enables the robot to weight sensory signals dynamically, suppress redundant or distracting information, and maintain appropriate behaviour under overstimulating conditions.

Regarding the RL model, we employ the Rescorla–Wagner model [20] to continuously evaluate and update the values associated with each stimulus, representing their dynamic importance for the robot. We have integrated the proposed system into a cuddly pet robot companion to perceive eight stimuli: touch in the head, ears, back, and belly, voice commands, Radio Frequency Identification (RFID) cards, and inertial movements (IMUs). The robot suffers from overstimulation during human–robot interaction due to its sensory richness and continuous false positives on its touch sensors due to electrostatic charge. We present three scenarios showing how integrating biologically inspired attention mechanisms enables intelligent sensor relevance and behaviour optimisation. These examples showcase the system’s ability to address key challenges in multisensory autonomous robots—such as overstimulation, perceptual redundancy, and stimulus prioritisation—and how it successfully mitigates specific issues detected in our robot during real-world interaction. In addition, we compare the robot’s response using the model to a baseline condition where the robot does not use these mechanisms to prioritise stimuli but stores associated expressions in a queue and performs them in order when possible.

This paper continues in Section 2, analysing the application of these mechanisms to other scenarios and perception systems to provide a theoretical basis for our approach. Section 3 defines the system and the RL method applied to obtain a dynamic adaptation to the perceived stimuli. Section 4 describes the pet robot companion and its characteristics. Section 5 describes the system’s evaluation methodology, including the experimental procedure and the metrics considered. Section 6 presents three case studies showing the application of the proposed methodology to a pet robot companion and compares the approach with a baseline method. Section 7 discusses the outcomes of the case studies and limitations of this work. Finally, Section 8 presents the conclusions and Section 9 the future work related to this research article.

## 2. Background

Perception systems are essential for representing the robot’s surroundings, organising data correctly, and filtering necessary information to facilitate and simplify the robot’s task. Some works propose perception systems based on biologically inspired mechanisms to improve stimulus processing in sensor networks. Within this context, habituation has been widely explored as a fundamental biological process that reduces responses to repeated stimuli, and several studies have investigated its computational modelling and application to robotics. For example, Mackintosh [21] proposed an attention mechanism in which learning processes dynamically regulate the allocation of attention, increasing sensitivity to stimuli that are more predictive of reinforcement. Similarly, Pearce and Hall [22] introduced a biologically inspired model where attention is modulated through prediction errors, allowing the system to adjust the relevance of each stimulus based on the discrepancies between expected and actual outcomes.

In the early work of Stanley [23], a computer simulation study of a circuit model of habituation based on Thompson’s two-process theory [24] was presented. The model simplified Thompson’s original circuit into direct and indirect stimulus–response pathways, with synaptic weights governed by differential equations to capture decremental and sensitisation processes. Simulations tested whether the model could reproduce empirically observed habituation curves, such as those from spinal cat reflexes. Results showed realistic response patterns across varying stimulus intensities and frequencies, demonstrating that declining sensitisation was not strictly necessary. The study concluded that computational modelling could provide functional insights into habituation and guide future investigations.

Building on such foundational work, Marsland et al. conducted two consecutive studies [25,26] that transferred habituation mechanisms to mobile robotics. They developed a neural architecture inspired by biological findings in toads and applied it to small autonomous robots equipped with light sensors programmed for phototaxis. Their experiments involved presenting steady, flashing, and dim light stimuli to assess whether the robots would gradually reduce their responses and whether responsiveness could recover when stimuli were altered or relocated. The evaluation measured response latency, habituation hierarchies, and locus specificity, drawing parallels with animal behaviour. The results confirmed that robots progressively ceased to respond to repeated stimuli, stronger stimuli took longer to habituate, and hierarchies emerged whereby certain stimuli suppressed reactions to others. Together, these studies demonstrated that habituation effectively filters repeated or irrelevant inputs in robots navigating dynamic environments.

Further applications explored habituation in more constrained scenarios. Chang [27] investigated sensor habituation in a Khepera miniature robot to improve navigation in narrow corridors. The study introduced a habituation mechanism into a neural network model of conditioning, using transmitter gates to reduce synaptic efficacy under prolonged stimulation. Comparative navigation trials in corridors of varying widths showed that habituation reduced oscillatory movements by over 90% in very narrow spaces while preserving obstacle avoidance and turning capabilities. This demonstrated that habituation could be integrated into neural controllers without retraining, improving the smoothness and stability during navigation.

Similarly, Cyr and Boukadoum [28] proposed a synaptic habituation learning rule within an Artificial Spiking Neural Network (ASNN) embedded in both simulated and physical autonomous robots. Their approach modelled the temporal modulation of synaptic responses to mimic natural habituation. It was tested in the AI-SIMCOG framework using Lego Mindstorms platforms. Experimental blocks exposed the robots to stimuli of varying intensity, duration, and stimulus intervals, assessing behavioural adaptations such as attenuation, recovery, and potentiation. Results showed exponential decay of responses, stimulus-specific adaptation, and the influence of stimulus characteristics on memory duration. The authors concluded that embedding habituation rules in ASNNs enhance adaptability by filtering redundant stimuli and supporting attentional focus in dynamic environments.

More recently, Gillard et al. [29] presented three computational models of habituation for embodied artificial agents, grounded in the Iterant Deformable Sensorimotor Medium (IDSM) framework. They proposed a sensor memory mechanism and two node-based penalty mechanisms to reproduce habituation and spontaneous recovery. These models were evaluated in simulation with repeated stimulation protocols, measuring behavioural trajectories, motor responses, and internal dynamics. Results showed that all models reproduced habituation and recovery under different conditions, with the sensor memory model excelling for continuous stimuli, while node-based mechanisms performed better for intermittent patterns. The study concluded that distinct computational strategies can implement habituation, each with advantages depending on stimulus characteristics, and that combining them may improve robustness. Recent advances in unsupervised image stitching [30] have integrated Generative Adversarial Networks with frequency-aware feature extraction in attention modulation through Convolutional Block Attention Modules and Transformer-based mechanisms. The model enables precise scene alignment with weak textures, low-light conditions, and significant parallax disparities.

Most studies on habituation have focused on sensorimotor adaptation, but related mechanisms also exist at the attentional level. IOR can be regarded as a form of habituation, and several works have explored it in computer vision as a strategy to filter perceived information. Hülse et al. [31] implemented IOR in a computer vision system to avoid paying attention to previously explored objects. Thus, the model successfully prioritises novel stimuli, reducing the computational load of the algorithm. The model consists of filters that improve image collection and a visual memory that remembers which objects the system has processed, reducing their importance. In a final step, the vision system moves and pays attention to novel stimuli, discarding those present in the visual memory. Heinen & Engel [32] presented a similar vision attentional model called NLOOK, intended for robotic vision systems. The model applies IOR to reduce the importance of visited regions in an image, focusing on new, unexplored areas. Thanks to this method, NLOOK improves computational demands compared to previous works, emphasising the positive effects of including biologically inspired mechanisms in artificial systems.

Vega et al. [33] presented a dynamic vision system based on visual memory and attention mechanisms for robot localisation. The system uses visual memory to collect relevant task-oriented objects and 3D segments to store information about the scenes the robot perceives. The attention module uses two object attributes, salience and “life”, to decide where to look at every moment and to forget objects when they disappear from the scene. Thus, the robot improves its exploration, computational load, and performance in service robot applications. Similarly, Chen & Tian [34] proposed visual attention for service robots based on a biologically inspired attention mechanism to improve the robot’s efficiency in perception and processing information. This mechanism predicts the focus of attention by fusing sensor data from different cameras to reduce the time to complete assistive tasks by prioritising relevant stimuli.

In line with the state-of-the-art works, our investigation group recently developed a bioinspired joint attention system designed to dynamically analyse and prioritise visual stimuli from both social cues (e.g., faces, gaze, gestures) and the environment (e.g., movement, light colour) in real time, which includes IOR [35]. This system grouped and ranked detected stimuli according to their estimated relevance, enabling the robot to focus on the most salient inputs over time. In a follow-up study, we implemented a responsive version of this system on the Mini social robot, allowing it to react to relevant visual stimuli during human–robot interaction scenarios. Results demonstrated improvements in social presence [36], competence, and warmth [37]. Continuing this line of research, we extend the attentional framework beyond vision to a multimodal setting and introduce the AF mechanism for managing perceptual overload in multisensor systems.

In summary, previous works have employed biologically inspired mechanisms such as habituation and IOR to regulate responsiveness in simulated agents and robotic systems, typically focusing on sensorimotor signals or filtering visual inputs to highlight novel or relevant stimuli. These studies underline the importance of attention mechanisms for reducing processing load by overlooking repeated information. Our contribution extends this line of research by shifting the focus to human–robot interaction in a companion pet robot, where multimodal and multisensory environments occur frequently and where stimulus repetition and overstimulation are well-known problems. Unlike previous approaches that mainly attenuate active signals, we introduce AF as a complementary mechanism to habituation and IOR, operating at the channel level so that overstimulated modalities are downregulated even when specific signals are inactive. The system has been integrated into the software architecture of the pet robot and is executed in real time, with the regulation of stimuli directly impacting the number of gestures produced and helps maintain expressive behaviour that is coherent and natural during interaction.

## 3. Bioinspired Attention System

This section defines the BIAS used to dynamically regulate the importance of the stimuli the robot perceives, justifying the algorithm we selected for our application. In addition, it shows how the model’s hyperparameters have been chosen to align with findings from human attention studies.

### 3.1. Computational Models

Different computational approaches have been proposed to adapt stimulus importance and attention. Classical habituation models typically use predefined rules that decrease attention depending on repeated stimulation and activation time [23,25,26]. These models modulate attention by capturing novelty through repeated exposure, but they are largely heuristic, not biologically inspired, and do not consider different types of stimuli. Similarly, Bayesian methods update the probability of stimulus relevance as new evidence becomes available [38,39]. They provide a framework that manages novelty. However, they depend on probability, without an explicit bioinspired basis, and a low adaptive response.

In cognitive neuroscience and psychology, RL is widely used to describe learning processes driven by reward prediction errors. Within this framework, the Rescorla–Wagner model can be understood as a special case of Temporal Difference learning, as discussed by Sutton and Barto [40], and further supported by authors such as Niv [41] and Dayan and Daw [42]. The Rescorla–Wagner model [20] has served to modulate attention with biologically inspired principles. They learn via prediction errors but do not explicitly handle novelty or stimulus persistence. Similarly, Mackintosh [21] and Pearce–Hall [22] models are bioinspired and modulate attention. Pearce–Hall also tracks novelty through prediction errors, while Mackintosh tracks it by applying prediction to modulate attention focus.

These six approaches can be applied to modulate attention in different ways, as Table 1 compares. We adopt a variant of the Rescorla–Wagner formulation because it offers (i) a direct connection to empirical models of learning in psychology and neuroscience, (ii) a computationally efficient update rule suitable for real-time robot perception and adaptation, and (iii) a principled grounding within RL frameworks based on prediction errors to consider novelty and persistent activation. Compared to habituation or Bayesian models, Rescorla–Wagner provides a positive balance between biological basis, computational adaptability, and extensibility toward more complex mechanisms.

### 3.2. System Design

The BIAS dynamically and simultaneously regulates the importance given to the stimuli the robot perceives using its sensors. The system’s input is perception data informing the system about the sensors’ activation. These data are used to define stimuli based on the following parameters, which are updated at each time step *t* of 0.6 s.

- **Name**: Unique name given to the stimulus.
- **Type**: Stimulus type. This value depends on the robot sensor that provides the information, such as touch, IMU, RFID or voice commands.
- **Data**: Raw perceptual value provided by the sensor. This content varies depending on the type of stimulus. For instance, it may correspond to a Boolean contact state for touch, a RFID tag ID, motion data from the IMU, or the recognised utterance in the case of speech recognition.
- **Status**: This parameter indicates whether the stimulus is active. It takes a value of 1 if the stimulus is active or 0 if it is inactive.
- **Last active time ($t_{last}$)**: Timestamp defining the last time the stimulus was active.
- **Active time ($t_{active}$)**: Set the number of seconds in float precision the stimulus has been active. If the stimulus is inactive, this parameter takes a 0.
- **Importance (*I*)**: Factor from 0 to 1 that represents how important the stimulus is for the robot in the current time step. Values close to 1 indicate that the stimulus is relevant in the current time step. Values close to 0 indicate that the stimulus is unimportant for the robot in the current time step.
- **Filtered Attentional Value (FAV)**: Value obtained from multiplying the stimulus importance by its status. This value creates a relevance ranking of all the stimuli the robot perceives.

Our system computes, at every time step *t*, the filtered attentional value given to each stimulus ($FAV_{s}$) using the parameters previously defined to create a ranking that sorts all stimuli by relevance. Equation (1) computes the stimulus relevance as a product of its *Status* (active 1 or inactive 0) by its importance $I_{s}$, a Q-value dynamically adjusted using RL for each stimulus *s*.

(1) $${\mathrm{FAV}}_{s}\left(t\right)={\mathrm{Status}}_{s}\left(t\right)\times I_{s}\left(t\right)$$

The model applies well-known RL methods in a robotics application to calculate the importance $I_{s}$ of each stimulus perceived by the robot. These importances are represented as Q-values, in analogy to value functions in RL. In our case, Q-values do not correspond to state–action returns, but instead encode each stimulus’s learned priority or salience, updated through a prediction-error rule. RL is a biologically inspired framework that allows an agent to learn adaptive behaviour from trial and error. It learns to prioritise and respond to stimuli by interacting with a changing world. Learning occurs by testing the benefits or drawbacks of executing a certain action under certain conditions, which is represented by a numerical reward. The agent performs an action, and if the action produces positive effects, the numerical reward will be high; if the action does not produce any positive effects, the reward will be low [43].

Many RL algorithms are available in the literature, as Sutton & Barto describe in their book [40]. This model uses a variation of TD(0) [44], a Temporal Difference algorithm that updates value estimates based on the difference between successive predictions. Unlike Monte Carlo methods, which wait until the end of an episode, TD(0) performs updates incrementally after each time step, making it suitable for online and real-time learning scenarios such as the attentional modulation proposed in this work. TD(0) estimates the state-value function $V^{\pi }\left(s\right)$ for a given policy π. Equation (2) shows the update rule of the state-value function.

(2) $$V\left(s\right)\leftarrow V\left(s\right)+\alpha \left[r+\gamma V\left(s^{\prime }\right)-V\left(s\right)\right],$$

where α is the learning rate that represents how fast the system adapts the estimated importance of a stimulus, the discount factor γ∈[0,1] represents the importance of future rewards over past rewards, *r* is the immediate reward received after executing an action, and V(s) and $V(s^{\prime })$ are values associated to states *s* and $s^{\prime }$.

Rescorla–Wagner updated the original update rule for TD(0) to calculate a new estimate based on the difference between the received reward and the current estimate. The rule adjusts the estimated value incrementally, moving it closer to the observed outcome [20]. This update is especially useful in psychology and cognitive modelling to represent reactive and adaptive systems, like the attentional system proposed in this work. Equation (3) describes the update rule proposed by Rescorla–Wagner, considering the application of our system. In our approach, states are the stimuli the robot perceives, and the importance factor $I_{s}$ encodes its dynamically computed attentional importance as the algorithm’s Q-value. These values do not converge to a final solution. Instead, they are continuously adjusted to represent the importance of the stimulus in each step. All stimuli start with the same importance value, set to 1 unit.

(3) $$I_{s}\left(t\right)\leftarrow I_{s}\left(t-1\right)+\alpha \left[r-I_{s}\left(t-1\right)\right],$$

where α is the learning factor from 0 to 1, *r* is the reward value computed by the reward function limited from 0 to 1, $I_{s}\left(t-1\right)$ is the importance factor in the current time step *t*, and $I_{s}\left(t-1\right)$ is the importance factor in the previous time step t−1.

The BIAS dynamically tracks the importance of all stimuli as a function of the IOR and AF mechanisms. These values are related by the reward function, which is used to compute the stimulus importance on each time step. Equation (4) shows the reward function designed to update the stimuli’s importance $I_{s}\left(t\right)$ depending on the AF and the IOR. As stated before, the reward value is limited from 0 to 1. Rewards close to 1 indicate the stimulus is relevant and its importance is rising. Rewards close to 0 indicate that the importance of the stimulus is dropping due to IOR and FA modulation.

(4) $$r_{s}={AF}_{s}\left(t\right)+{IOR}_{s}\left(t\right)$$

IOR dynamically reduces the salience of stimuli that remain active over time. We define a time-dependent function that decreases the importance of a stimulus as a function of how long it has remained active. Specifically, we propose Equation (5) to compute the IOR applied to each stimulus *s* at time step *t*. The equation depends on $w_{ior}$ to adjust the sensitivity of the inhibition curve. A smaller value of $w_{ior}$ causes inhibition to rise more quickly, while a larger value produces a more gradual decline in salience. This flexibility allows the system to show different attentional dynamics depending on the task or environment.

(5) $$IOR_{s}\left(t\right)=-\frac{t_{\mathrm{active}}}{t_{\mathrm{active}}+w_{ior}}$$

Similarly, AF reflects the progressive decline in priority assigned to persistently or repeatedly activated stimuli within the same sensory modality or functional category. In our system, AF operates as a cumulative and generalising mechanism, discouraging over-reliance on specific input channels and promoting perceptual diversity. We propose Equation (6) to compute the AF of a stimulus *s* at time step *t*.

(6) $$AF_{s}\left(t\right)=1-\sum_{i=1}^{N}\frac{t_{\mathrm{active},i}\left(t\right)}{t_{\mathrm{active},i}\left(t\right)+w_{a\;f}}$$

This formulation captures the idea that prolonged or repeated activation of a given sensory modality reduces attentional priority for that category. As the number of stimuli of the same type increases, the denominator in each term becomes larger, reducing the overall contribution of each term in the summation and thereby decreasing AF. As a result, stimuli from overused sensory channels are dynamically reduced in importance in favour of others. The AF only applies if there is more than one stimulus of the same time active (e.g., different touch sensors activated simultaneously). Otherwise, the AF equals 0. In this equation, *N* is the number of active stimuli of the same type, $t_{active,i}$ is the time for which the stimulus *i* has been active, and $w_{a\;f}$ adjusts the system’s sensitivity to fatigue accumulation. Lower $w_{a\;f}$ values result in faster penalisation for repeated stimuli, while higher values allow for more tolerance before attentional priority is reduced. The values of IOR and AF have been constrained within the range [0,1] to prevent fast system responses and to ensure smoother attentional modulation.

The output of the BIAS is a ranking sorting all the stimuli the robot perceives by relevance, with scores from 0 to 1. Stimuli with higher relevance scores are more important for the robot than those with lower scores, whose relevance has been reduced due to a long-time activation (IOR) or the overstimulation of sensors of the same modality (AF).

### 3.3. Parameters Selection

The system’s performance depends on three parameters that influence the speed at which the stimulus importance changes: α, $w_{ior}$ and $w_{a\;f}$. The learning rate α=0.3 regulates the adaptation speed of stimulus importance over time. The parameters $w_{ior}=0.015$ and $w_{a\;f}=30$ have been selected to control the rate at which inhibition and fatigue occur–allowing IOR to respond quickly to recent activations and AF building on a gradual decrease across stimuli of the same category.

These parameters were selected by running different simulations. In each simulation, two parameters were constant while the third was varied to evaluate its effects and identify a value that meets the system’s requirements. We followed bioinspired conditions from human neuroscience studies to guide parameter tuning as follows:

1. Ensuring the stimulus importance drop due to inhibition occurs within 0.2 to 2 s [45]. We decided to use this value to give our system enough time to receive and process all stimuli, since some messages were lost with lower values.
2. Ensuring the importance drop due to fatigue in inactive stimuli occurs from 30 s to 2–3 min [46]. We decided to set the loss of importance due to Attentional Fatigue to 30 s to allow fast responses in our robot, decreasing the importance of sustained stimuli.

**Figure 1:**
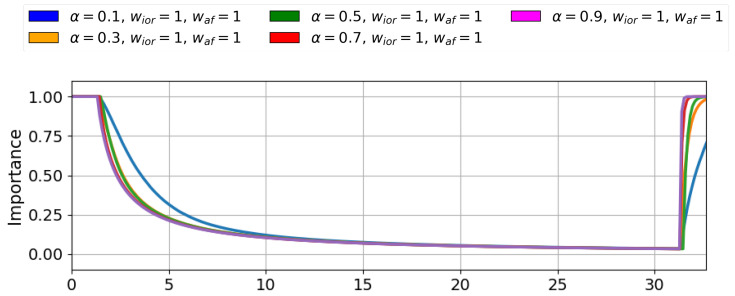
Q-values defining the stimulus importance for different α values. Lower α produce slower adaptive response, affecting the system reactiveness to novel stimuli. On the other hand, Higher α values produce very fast responses, occasionally leading the robot to miss relevant information.

First, we set the $w_{ior}$ and $w_{a\;f}$ values to 1.0, and ran a simulation to find an optimal α value. Each simulation consisted of activating a stimulus for 30 s and observing the time and slope of the importance drop. As α ranges from 0 to 1, the values tested were: 0.1, 0.3, 0.5, 0.7, 0.9 to analyse their dynamic decay. Figure 1 shows the results obtained in the simulation. The plot highlights the influence of the parameter α on the importance drop rate. The higher the value of α, the faster the importance decreases.

Table 2 shows the quantitative results of the simulations run to select parameter α. The table includes the fall duration, defined as the time it takes for the stimulus importance to decrease from 1 to 0 once it becomes active, and the fall slope, which indicates the rate of this decline. As none of the tested values satisfied the condition of producing a importance drop in less than 2 s and different α values do not show significant variations among them, we selected the α value of 0.3 to avoid abrupt changes in the stimuli’s importance between time steps and focus on variating the other two parameters, $w_{ior}$ and $w_{a\;f}$.

The next parameter to adjust was $w_{ior}$, as it could help achieve the desired importance drop rate, which was not met through α selection alone. The simulations to tune $w_{ior}$ were conducted with α=0.3 and $w_{a\;f}=1$, activating only one stimulus and reducing its activation time to 8 s to showcase the dynamics and differences among options. Although tests were performed with values across a wide range of magnitudes, only the values close to 2 s are shown.

**Figure 2:**
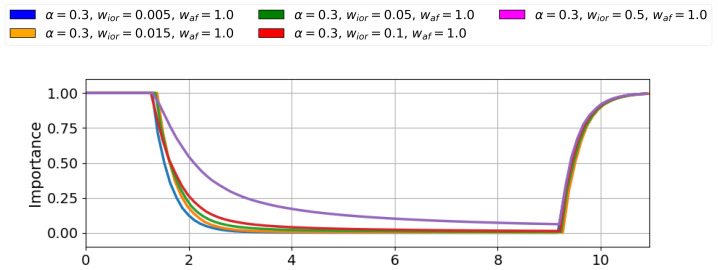
Q-values defining the stimulus importance for different $w_{ior}$. Lower $w_{ior}$ values provide faster adaptive response in the system while higher values produce slower adaptation.

Figure 2 shows the simulation results for tuning the parameter $w_{ior}$. The plot illustrates the inverse relationship between $w_{ior}$ and the importance decay speed; the lower the $w_{ior}$ value, the faster the importance drops. Table 3 presents the quantitative results for the $w_{ior}$ simulations. A significant decrease in $w_{ior}$ satisfies the condition of producing the importance inhibition in a time below 2 s. The value that best meets this requirement is 0.015, which was therefore selected as the final value.

The last parameter, $w_{a\;f}$, was selected fixing α=0.3 and $w_{ior}=0.015$. The simulations involved activating two stimuli of the same type for 40 s and observing the influence on the importance of an inactive stimulus of the same type.

**Figure 3:**
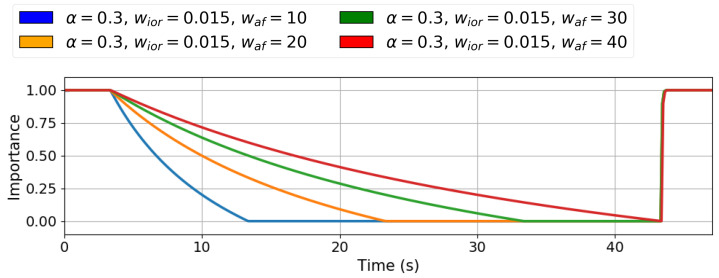
Q-values defining the stimulus importance for different $w_{a\;f}$. Higher $w_{a\;f}$ values make the stimulus importance decay slower. On the other hand, lower $w_{a\;f}$ values provide higher importance decay rates.

Figure 3 shows a plot with the simulation results for the $w_{a\;f}$ tuning. The graph illustrates the relationship between the $w_{a\;f}$ value and the time the importance of stimulus changes from 1 to 0. Table 4 presents the quantitative results for the $w_{a\;f}$ tests. To satisfy the system’s second requirement of having a dropping importance duration of at least 30 s for inactive stimuli, the chosen value for the $w_{a\;f}$ parameter is 30. It is worth noting that, with the AF influence on the active stimuli with this value, the importance dropping time is amplified in the active stimuli and reduced to 1.87 s, but still satisfies the system’s predefined conditions.

## 4. Pet Robot Companion

**Figure 4:**
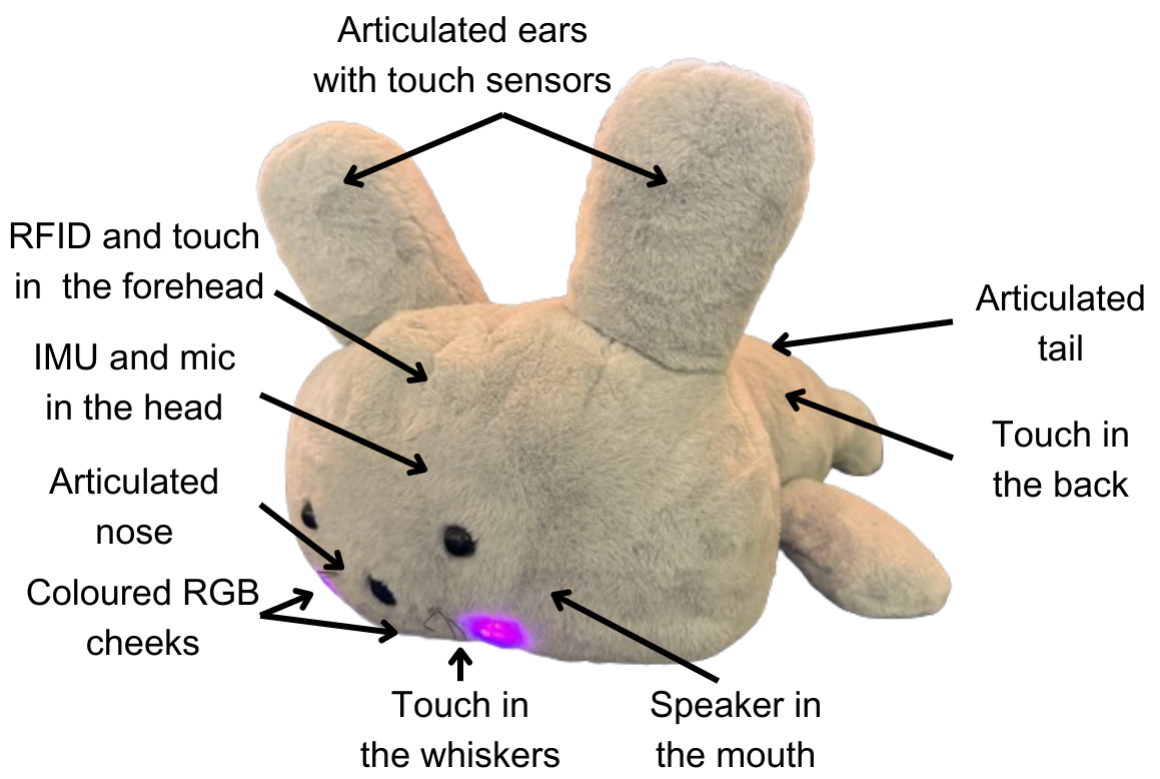
Our pet robot companion, with its sensors and actuators highlighted.

**Figure 5:**
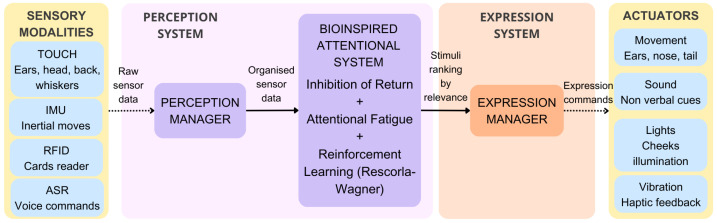
Diagram showing the software architecture of the pet robot companion that integrates the BIAS designed to regulate stimuli relevance.

Our pet robot was conceived to provide companionship and affection to older adults who feel alone. As Figure 4 shows, the robot has a friendly animal appearance endowed with different sensors that enable a reactive behaviour responding to the stimuli it perceives using a ROS 2 modular software architecture. Figure 5 shows a diagram representing the software architecture that defines the reactive behaviour of our pet robot companion. The BIAS is situated in the centre, regulating stimulus relevance.

The robot perceives ambient stimuli using the following four different modalities: touch, using five capacitive tactile sensors located in its ears, whiskers, head, and back; voice commands, using ambient sound captured by a microphone that is translated and filtered using an offline Automatic Speech Recognition (ASR) system using a custom word dataset with Vosk engine [47]; RFID cards, using a RFID reader located in its forehead; and movements. using a 9 degrees of freedom IMU that provides orientation, angular velocity, and angular acceleration along three axes.

A Perception Manager receives the raw information captured by these sensors [36], a module that processes this information and generates, for each modality, a unified message that keeps track of the sensors’ activation. This message is received by the BIAS presented in this paper, which applies the IOR and AF combined with RL to manage sensor overstimulation and repeated activation. The BIAS generates a ranking sorting stimuli with higher importance above.

The Expression Manager receives the output of the BIAS and translates the ranking of stimuli into expressions that the robot executes. Our pet robot companion executes purely reactive behaviour, matching each stimulus with a predefined expression, as Table 5 shows. To perform these expressions, the robot has four degrees of freedom in the nose, ears, and tail, luminous devices in the cheeks that can be illuminated in different colours, a vibration motor to provide haptic feedback to the user, and a speaker to play non-verbal sounds. The robot only executes one expression at a time, reacting to the most relevant stimulus, plus a respiration movement using the motor in the nose. For example, suppose the robot detects that the user is touching one of its ears. In that case, the robot will perform an expression that simulates joy by illuminating its cheeks in orange, moving both ears, activating the vibrating motor, and purring.

## 5. Experimental Setup

The system evaluation consists of integrating the bioinspired architecture into the pet robot companion presented in the previous section to regulate the stimuli it perceives. The following sections describe the experimental procedure and the metrics used to present the most relevant results.

### 5.1. Procedure

The tests illustrate the operation of the proposed attentional mechanisms (IOR and AF) and assess the system’s behaviour under complex multisensory conditions compared to a baseline approach. First, we present three case studies highlighting a specific aspect of the system’s operation. Each test is accompanied by a narrated description of the events occurring during the interaction to provide context for the system’s perceptual and attentional response.

For every demonstration, we present the following three types of visualised data over time: (i) the raw perception signals captured by the robot’s sensors, (ii) the Q-values computed by the RL algorithm computed using the IOR and AF mechanisms, (iii) the reward value evolution, and (iv) the filtered attentional value, which results from multiplying the Q-value by the stimulus activation status. With this information, we compare the original sensory input with the corresponding Q-values and filtered outputs. This allows us to observe how the system transforms and regulates incoming signals once the attentional mechanisms are applied. In doing so, we can evaluate the behaviour of the RL model in real time and assess whether it aligns with the expected dynamics of the BIAS.

Secondly, we present the benefits of the robot’s expressiveness in the proposed systems by comparing its performance with a baseline. These approaches differ in how the robot processes the stimuli and performs its expressions. To conduct this experiment, we use the input information presented in the third case study to calculate some metrics that compare the previous system in the robot with the BIAS. The working conditions of the BIAS and the Baseline System are the following:

- **Baseline System:** The robot receives input data and does not perform any processing, directly sending the stimuli it perceives to the Expression Manager. The Expression Manager matches the stimulus with an expression (as Table 5 shows) and stores the list of expressions the robot has to perform, sorted by arrival time, in a queue. When there is an expression in the queue, the robot executes it. Once finished, it performs the following order until no expressions are in the list.
- **Attentional Biosinpired System:** The robot receives input data, and the proposed system filters the information, generating a ranking of stimuli where the most important ones appear first. The Expression Manager receives the ranking, matches the most important stimulus with its corresponding expression, and stores the expression in a queue. This step is carried out every time the most important stimulus changes and has an importance value above 0 units. Then, as in the Baseline condition, the Expression Manager performs the expressions stored in the queue one by one until no more expressions are available.

### 5.2. Evaluation Metrics

The comparison employs different evaluation metrics that analyse the expression selection and execution in both systems. To facilitate their comprehension in the results section, we describe their meaning as follows:

- **Expressions in the queue:** This metric represents the average number of expressions that the robot has in the queue during the experiment. If the Expression Manager receives many stimuli due to overstimulation, the number of expressions will increase, leading to repeated behaviours.
- **Time to completion:** Represents the average time the Expression Manager takes to start executing an expression since its reception. This metric is related to the average expressions in the queue since the higher the number of expressions in the queue, the higher the delay in performing them.
- **Computational consumption:** Represents the computational consumption by the Expression Manager. It represents the consumption percentage of one core of the robot’s computer.

## 6. Results

The results of this study are divided into two parts. First, we present three case studies showing the system’s performance. Second, we show the improvements produced by the system, comparing the robot’s expressiveness with and without the BIAS.

### 6.1. Case Studies

The first case explores the isolated effect of IOR by observing how the robot reduces the relevance of a continuously active stimulus, more specifically, the RFID sensor on its forehead. The second demonstration focuses on the role of AF when several stimuli of the same type are activated simultaneously, showing how the system prevents overstimulation by modulating the attentional value of three touch sensors located on the head, whiskers, and back. Finally, we demonstrate the system’s full functionality under complex multisensory conditions in a real interaction with a user, where the robot reacts to touch, motion, RFID cards, and speech. This scenario illustrates how the system prioritises newer or more salient stimuli and adapts the robot’s responses accordingly.

#### 6.1.1. Managing Persistent Stimuli Through Inhibition of Return

**Figure 6:**
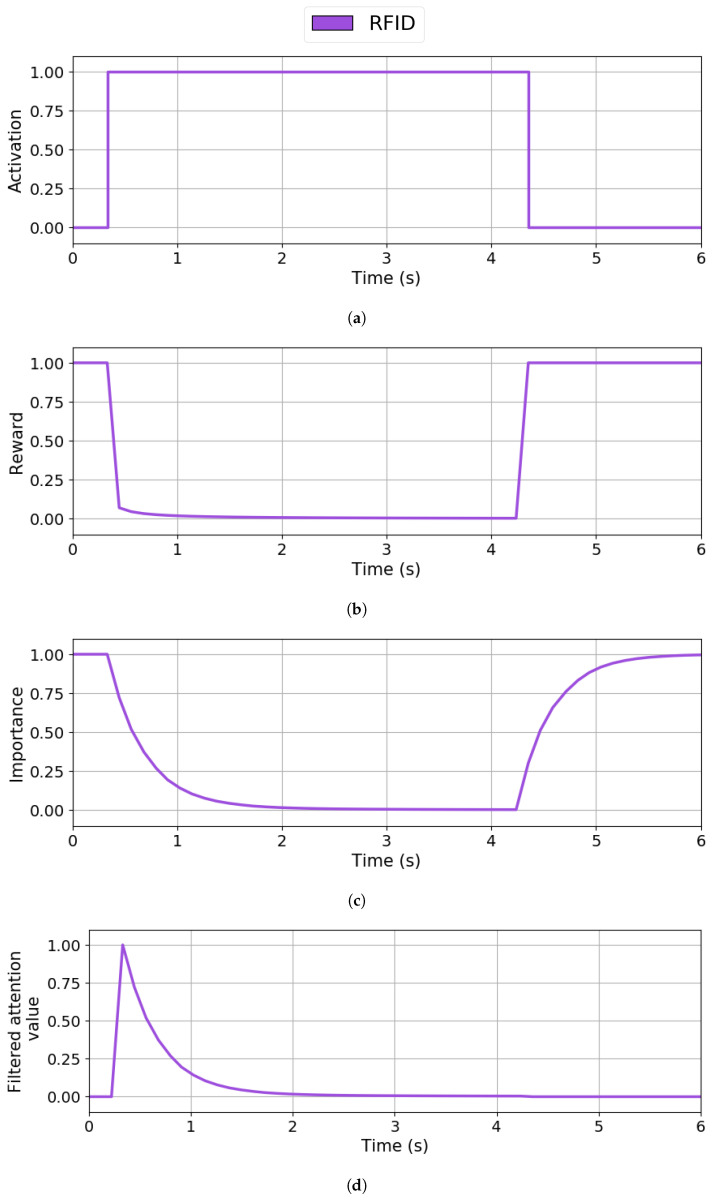
Inhibition of Return effects on RFID reader stimuli. (**a**) System input defining sensor activation. (**b**) Reward value obtained from applying the IOR. (**c**) Q-values defining the stimulus importance. (**d**) System output defining the filtered attentional value.

The IOR dynamics are demonstrated in Figure 6, using a scenario where the user interacts with the pet robot companion by passing a RFID card over his forehead for 4 s, leading to sustained activation of the RFID perception channel. Such prolonged stimulation causes the robot to persistently focus on this single input without attentional regulation, potentially ignoring other relevant stimuli. This demonstration aims to observe how the IOR mechanism progressively reduces the relevance of this persistent input over time.

Figure 6a shows the stimulus activation, which is the system input provided by the robot sensors. At approximately t=0.4 s, the RFID reader becomes active due to detecting a RFID card. Four seconds later (t=4.4 s), the stimulus changes to inactive.

Figure 6b shows the reward provided to the RL system, representing the desired stimulus importance values that guide its dynamics. As soon as a stimulus becomes active, the reward drops almost instantly to zero due to the influence of the IOR mechanism; once the stimulus deactivates, the reward returns to one since no attention mechanisms are applied.

Figure 6c shows the importance of the stimulus calculated by the RL system. According to this graph, when the stimulus becomes active, its importance starts dropping due to the influence of the IOR mechanism. The RFID reader stimulus loses importance with time with a slope close to 0.476 per second, reaching the lowest limit of 0.0 units at t=2.4 s. At this point, the inhibition applied to the stimulus remains until the sensor changes to the inactive status at t=4.4 s. Once the stimulus is inactive, the system starts restoring importance to its default value of 1.0 unit with a speed of 0.830 units per second.

Figure 6d shows the system output regarding the stimuli’s filtered attentional value. The system considers the RFID reader stimulus the most relevant as it is the only active one with a filtered attentional value of 1.0 units at t=0.4 s. As the stimulus loses importance over time due to the IOR, it keeps grabbing the robot’s attentional focus since it is the only stimulus active. However, once the IOR mechanism completely inhibits the stimulus, the robot completely loses interest in the stimulus and stops considering the stimulus as relevant. The filtered attentional value does not show the stimulus recovery since the stimulus is inactive.

#### 6.1.2. Reducing Redundant Inputs with Attentional Fatigue Mechanism

**Figure 7:**
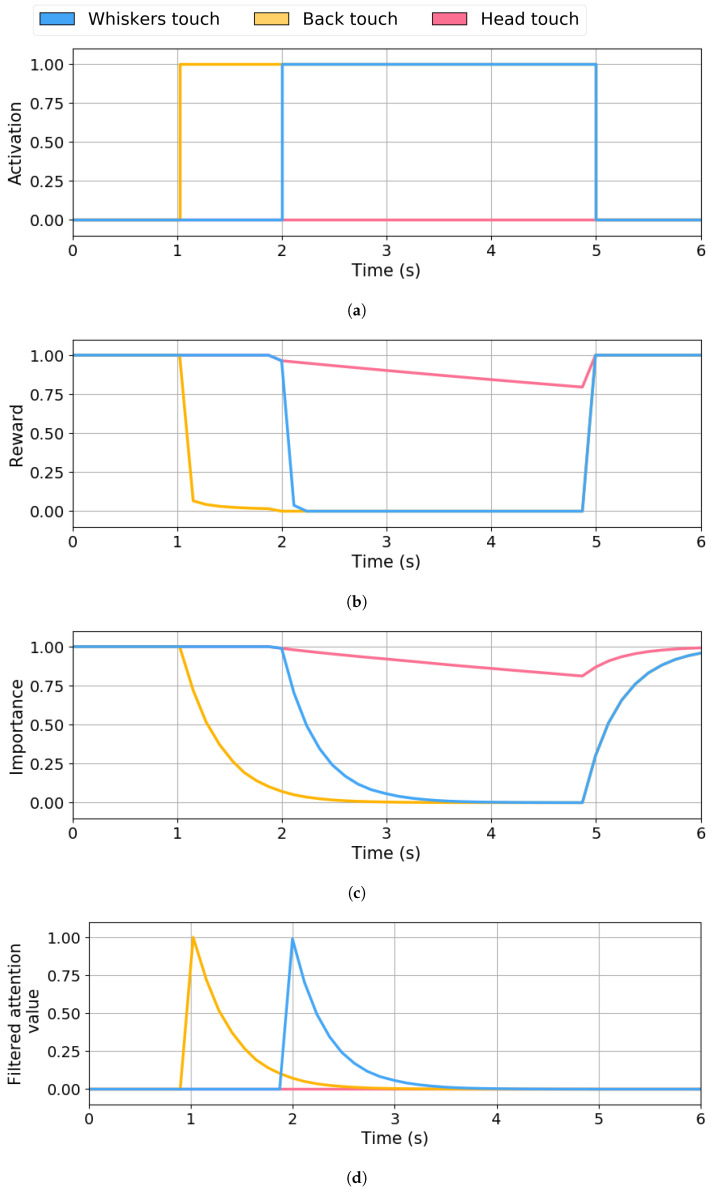
Attentional Fatigue produced on touch stimuli that occur at the same time interval. (**a**) System input defining sensor activation. (**b**) Reward value obtained from applying the IOR and AF. (**c**) Q-values defining the stimuli’s importance. (**d**) System output defining the filtered attentional values.

The AF dynamics are demonstrated in Figure 7 with a scenario where the user touches the pet robot’s back and touches its whiskers after 1 s. After 3 s, the user ceases both stimulations. Both interactions belong to the same sensory channel, touch, which is particularly prone to overstimulation in social robots. Without attentional regulation, simultaneous activations of multiple touch sensors result in ambiguous or redundant behaviours, as the robot might attempt to respond equally to each contact. This demonstration is designed to show how the AF mechanism reduces the importance of all stimuli of the same type, regardless of whether they are active. Although only the tactile channel is illustrated in this example to isolate and highlight the behaviour of the AF mechanism, the primary purpose of this mechanism is to promote a shift of attention away from overstimulated modalities and allow the system to focus on other, potentially more relevant sensory channels. While the focus here is on the effects of AF, it is worth noting that the IOR mechanisms remain active in the background, as they are inherent in the RL algorithm.

Figure 7a shows the stimuli activations as the system’s input. The first activation occurs at t=1 s, when the back touch sensor (yellow line) becomes active. One second later, at t=2 s, the whiskers’ touch sensor (blue line) status changes to active. Both stimuli remain active simultaneously for three seconds, until t=5 s.

Figure 7b shows the reward given to the RL system to adjust the importance of the stimuli. At t=1 s, as the back touch (yellow) activates, its reward decays to zero across two distinct phases due to the IOR mechanism as follows: an initial sharp decline with a slope of around 9 points, followed by a slower decay phase with a slope of 0.111, finally reaching the 0.0 units at t=2 s. However, once the whiskers’ touch (blue) becomes active, at t=2 s, the AF mechanism starts to influence the reward of all the stimuli of the touch type, as two stimuli of this type are active simultaneously. Its influence can be perceived in the following: the whiskers’ touch reward (blue), as the second slope observed with the back touch stimulus becomes faster (slope of 1), and the inactive stimuli, in this case, the head touch (pink line), where reward starts decreasing gradually (slope of 0.083). Once the two stimuli become inactive again, the reward returns rapidly to one (slope of 10).

Figure 7c shows the evolution of the stimuli’s importance computed by the RL system. The importance dynamics follow the reward tendencies; however, it has a smoother changing slope. The back (yellow) and whiskers’ (blue) touch stimulus has a dropping slope of 0.571 units per second, reaching 0.0 units at t=2.75 s and t=3.75 s, respectively. Meanwhile, touch-type stimuli (pink) keep the same speed as their reward. The stimulus importance recovery to 1.0 units starting at t=4.9 s is also slower than the reward, with a slope of 0.91 for the active stimuli (overlapped by the blue line) and 0.23 for the inactive (pink).

Figure 7d shows the filtered attentional value of each stimulus as the system output. At t=1 s, the most relevant stimulus is the back touch with a value of 1.0 units. This stimulus remains the robot’s attentional focus until, at t=2 s, whiskers’ touch becomes active. As the whisker touch has a value of 1.0 units, which is greater than the back touch value (0.1 units), this stimulus becomes the new robot’s attentional focus. Once the system inhibits the whisker touch stimulus, the robot loses interest and considers no stimuli relevant to its attention.

#### 6.1.3. Real-Time Stimulus Prioritisation in a Multisensory Scenario

**Figure 8:**
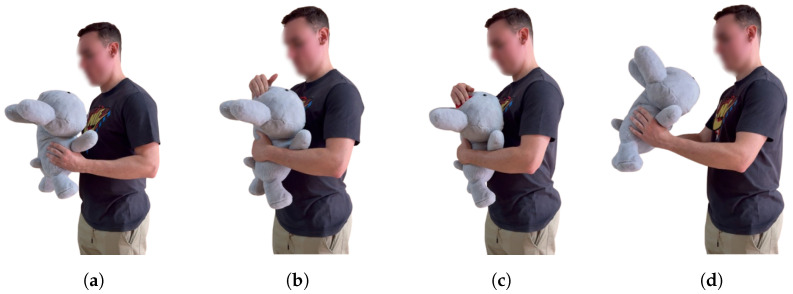
Scenes showing the user interacting with the robot and activating different sensors. (**a**) The user greets the robot activating its ASR. (**b**) The user touching its back and head. (**c**) The user passes a RFID card on its head. (**d**) Robot rotation activating its inertial sensor.

**Figure 9:**
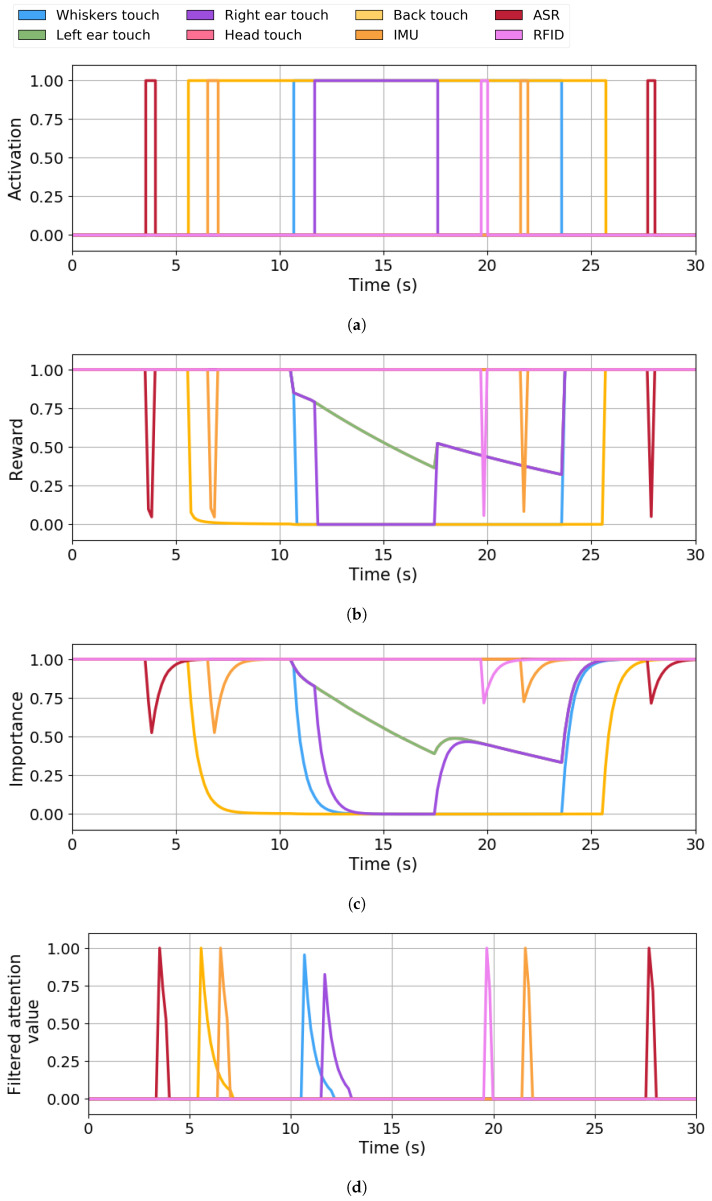
BIAS applied to multiple stimuli. (**a**) System input defining sensor activation. (**b**) Reward value obtained from the IOR and AF. (**c**) Q-values defining the stimuli’s importance. (**d**) System output defining the filtered attentional values.

The last case shows a real interaction between the robot and the user (with scenes shown in Figure 8) involving activating multiple sensors of different modalities. The proposed scenario is an interaction where the user interacts with the robot through various channels. This case shows the stimulation of the following four different types of stimuli: speech detection using an ASR, touch contact in other parts of the robot, using RFID cards acting as objects the robot needs, and inertial movements (IMUs). The duration of this interaction is 30 s. Without attentional regulation, the robot would attempt to respond equally to all incoming signals, regardless of their relevance, modality, or novelty, potentially resulting in overloaded or incoherent behaviours when multiple stimuli overlap or compete. With the proposed system enabled, the combined effect of IOR and AF mechanisms is expected to help the robot modulate its attention across modalities, promoting a more selective and organised stimulus processing. Figure 9 contains the graphs of the BIAS managing the perception information during this interaction.

Figure 9a shows the sensors’ activation. The robot starts idle, so no stimuli are active. The user greets the robot by voice, and the ASR (red line) activates (t=3.5 s). Then, the user grabs the robot, and the back touch sensor (yellow line) becomes active (t=5.5 s). Since the user holds the robot touching the back, this sensor remains active until t=25.5 s. At t=6 s, the user moves the robot, and the IMU (orange line) changes to active for half a second. At t=10.5 s, the user starts stimulating the robot’s whiskers (blue line) with the same arm as he holds the robot, maintaining the touch in the robot’s back with his upper arm. At almost t=11 s, the user unintentionally touches the robot’s right ear with its forearm for six seconds (t=17 s). Later, at t=19.5 s, the robot detects a RFID card (violet line) provided by the user using the hand not employed in holding the robot. The user moves the robot again at t=21 s, instantly causing the IMU (orange) to become active. The whiskers (blue) and back (yellow) touch values change to inactive at t=24 s and t=25.5 s, respectively, as the user stops stimulating and holding the robot. Finally, the user says goodbye to the robot, so the ASR (red) becomes punctually active at the end of the interaction (t=27.5 s).

Figure 9b shows the reward value obtained from the IOR and AF mechanisms. The reward of the stimuli that becomes punctually active, such as the ASR (red), IMU (orange), and the RFID reader (violet), changes to 0.1 units for an instant and then gets back to 1.0 units. This happens due to the short amount of time the stimulus becomes active. Meanwhile, the reward of the prolonged active stimuli drops quickly to zero once they change to active, and it quickly recovers to one as soon as they deactivate. This happens with the stimuli of the touch channel, as follows: back (yellow), whiskers (blue) and right ear (purple). On the other hand, among the inactive stimuli, only those from the touch channel (overlapped by the green line) have their reward affected by the AF mechanism from t=10.5 s to t=24 s caused by the simultaneous activation of two or more touch stimuli. The drop in their reward begins at t=10.5 s, indicated by the purple line (which overlaps the inactive touch stimuli) with a slope of 0.033 units per second. This continues until the third touch-type stimulus, the right ear (purple), activates at t=11; at this point, the slope increases to 0.063 units per second. Once this stimulus changes to inactive again (at t=17 s), the inactive touch-type stimuli (overlapped by the purple line) recover slightly and start dropping with the same slope of 0.033.

Figure 9c shows the dynamic evolution of the stimuli’s importance assigned by the RL system. The IOR dynamics affect all stimuli when they become active, making them lose importance with time and recover their value when they become inactive. This situation occurs at the beginning of the demonstration (t=3.5 s) with the ASR stimulus (red) and at the end of the interaction (t=27.5 s). Similarly, IOR effects can be perceived on the RFID (violet) at t=19.5 s and on the IMU (orange) at t=6 s and t=21 s. Touch stimuli follow similar dynamics due to the effects of the IOR, but the AF influence makes them lose importance when more than one touch stimulus is active. At t=5.5 s, the back touch (yellow) loses importance as it becomes active. It reaches the limit value of 0.0 units at t=9 s. At t=10.5 s, the whiskers touch (blue) changes to active and its importance drops until it reaches zero units at t=12.5 s. At the same time, the inactive touch-type stimuli (overlapped by the purple line) also lose importance, but at a slower speed, since only the AF is applied. Once the right ear touch (purple) changes to active, its importance drops faster than the inactive touch-type stimuli (green) due to the simultaneous action of the IOR and AF. When the right ear touch deactivates (at t=17 s), IOR is no longer applied, so its importance recovers until it matches the penalty speed of the inactive touch-type stimuli (at t=19 s). The same recovery dynamic happens with the whiskers touch sensor (blue) at t=24 s when it changes to inactive, joining the inactive touch-type stimuli. Finally, at t=25.5 s, the back touch deactivates and starts recovering its importance.

Figure 9d shows the filtered attentional value of the stimuli as the system output, which is the product of the importance value by the status defining whether the stimulus is active. The first stimulus the robot focuses on is the user’s voice (ASR, red line) at t=3.5 s. Then, the focus changes to the back touch stimulus (yellow) at t=5.5 s. At t=6 s, as the IMU (orange) becomes active, this stimulus takes the robot’s attentional focus as its filtered attention value (1.0 units) is greater than the back touch (0.15 units). This case highlights how a new perceived stimulus (IMU) takes the robot’s focus away from other stimuli (in this case, the back touch) that have lost importance with time. Immediately after the IMU (orange) deactivates (t=7 s), the back touch still has 0.1 units of filtered attention value, so it gains the robot’s attention again. At t=10.5 s, the primary focus changes to the whiskers’ touch (blue), with a filtered attention value of 0.95 units due to the AF influence. At t=36 s, the right ear (purple) becomes the robot’s attentional focus, with a filtered attention value around 0.80 units, surpassing the whisker’s touch (blue), which is close to 0.20 units. This scenario shows again how the right ear touch takes the robot’s focus over the whiskers touch due to the combined effect of the IOR and especially of the AF (since the right ear and whiskers’ touch become active while the back touch is active). Later, at t=19.5 s, the RFID (violet) becomes the new robot’s focus. At t=21 s, the robot focuses on the IMU (orange). Lastly, the ASR becomes the attentional focus again at t=27.5 s since no other stimuli are active.

### 6.2. Comparative Analysis

The case study presented in the previous section has been used to compare the robot’s expressiveness with and without the BIAS. This approach aims to show the improvement in the robot’s behaviour with the BIAS. With the BIAS, stimuli are filtered, avoiding repeated expressions and giving importance to novel and relevant stimuli. In the Baseline condition, stimuli are not filtered, causing repeated and delayed behaviours that accumulate in the Expression Manager. As shown in Figure 9d, eight peaks are associated with the activation of the stimuli, so the robot should perform the same number of expressions to respond to the user’s interaction. Table 6 shows the comparison outcomes regarding the following three evaluation metrics: time to completion, expressions in the queue and computational consumption.

The total number of expressions performed by the robot with the Baseline System is 85, mainly due to the prolonged activation of the touch sensors that lead to the repeated accumulation of the same expression in the queue. Meanwhile, with the BIAS, the robot only performs nine expressions. Eight of the nine expressions correspond to the eight stimuli with a significant importance level. The other expression is a repeated expression associated with the touch back sensor. This stimulus gains twice the robot’s focus at t=5.5 s. The first time is due to its novelty, but the second occurs after the IMU deactivation at t=7 s. At this moment, the importance of the touch back stimulus is low (close to 0.1) but still positive, leading the robot to repeat the expression.

On average, the Baseline System has more than 38 expressions in the queue while the experiment lasts. With the BIAS, the average number of expressions in the queue is subtly above 1. The Baseline System requires, on average, more than 128 s to execute the expressions since it queues a high number of them. The accumulation of expressions in the queue causes important delays in expression execution, making the robot perform reactive behaviours that must be done on time significantly later than expected. This value is reduced to 8.31 in the SABI condition, a positive time since all expressions take at least between 2 and 6 s to be performed. Finally, we supervised how much computational power the systems require to run each algorithm. The SABI requires more computational consumption (6.38% of one core) while the Baseline System is lighter, requiring only 1.38% of one core.

## 7. Discussion

This section summarises the main results of the study, provides an outlook of the most important events detected in the robot, and enumerates the limitations of the BIAS.

### 7.1. Summary

The results shown in the case studies illustrate the system’s capacity to modulate the importance of incoming stimuli in real time, effectively replicating the cognitive mechanisms it is inspired by. Although these case studies do not constitute formal validation with end-users, they demonstrate and analyse the system’s behaviour under different real conditions of multisensory stimulation.

In the first case, the robot was exposed to a single stimulus that remained active over an extended period. The integration of the IOR mechanism allowed the system to suppress the influence of this stimulus over time, preventing the accumulation of redundant responses and enabling the robot to remain attentive to new changes in the environment. This aligns with the biological function of IOR and confirms the system’s ability to reduce the weight of persistent inputs without manual filtering.

In the second case study, multiple stimuli of the same modality—namely, several simultaneous touch interactions—were activated. The introduction of AF in the reward structure led the system to penalise inputs from overstimulated modalities, reducing the chance of multiple responses being triggered from similar sources. This demonstrates how the system can help maintain response coherence when a specific perception channel is saturated or subject to repeated interactions, promoting a shift of attention to focus on other sensory channels.

The third case presents a more complex scenario involving multiple stimuli from different sensory modalities (touch, IMU, RFID, and speech). The system dynamically balances the importance of these inputs based on their novelty and modality saturation. Recent or isolated stimuli were favoured over those that had remained active or belonged to overstimulated channels, resulting in more reactive and contextually adequate behaviour. This case study shows the system’s scalability and ability to operate under realistic, multimodal conditions while preserving a consistent attentional focus.

Finally, the comparison between the BIAS and a Baseline System that was previously working in the robot shows the positive effects in stimulus management produced by the BIAS. With the BIAS filtering stimuli by importance using IOR and AF mechanisms, the number of expressions performed by the robot drops from 85 to 9, causing a significant decay in the delay to be executed and the average number of expressions in the queue during the experiment. However, the BIAS requires more computational power since the algorithm applied to compute importance contains more operations.

### 7.2. Results Analyses

This work demonstrates that biologically inspired attentional behaviours can be combined and applied with RL to provide robots with adaptive behaviour. Rather than implementing attentional mechanisms as static rules or predefined modules that previous studies addressed [21,22,23], we have encoded their principles into the reward function of the RL algorithm. This approach allows the robot to regulate attention adaptively based on experience and novelty, producing meaningful outcomes such as novelty preference, stimulus downregulation, or redundancy suppression. By doing so, the system offers a simultaneous, unified, and adaptive strategy for managing multisensory input in real time, bridging the gap between neurocognitive inspiration and computational implementation.

In addition to its conceptual design, the proposed system has been implemented in a pet-like robotic platform and tested in various real-world case scenarios. They demonstrate the feasibility of embedding biologically inspired attentional regulation into a functional, sensor-rich platform. Previous approaches [26,31] that regulated robot attention only focused on one perception type instead of multisensor domains, not considering the broad number and overlapping perception channels existing in living organisms. Our system mitigates issues frequently observed in tactile and multisensory interactions, such as redundant behavioural responses (similar to Marsland et al. [25]) and uncontrolled stimulus chaining. These limitations were previously discussed by Nunez et al. [11] and encountered in our investigation group’s earlier work with this platform.

The results show a clear improvement with the BIAS regarding the queue length and the time to completion metrics. The length of the expression queue was significantly reduced due to the dynamic stimuli regulation provided by the BIAS. In addition, the delay in the expressions caused by accumulating them in the queue is much lower with the BIAS than in the previous software, which only performed expressions without filtering stimuli by importance. Regarding computational consumption, the BIAS slightly utilises more resources due to the algorithm load. However, the computational load remains sustainable and does not compromise the robot’s operation.

### 7.3. Limitations

The evaluation of this system has reported some limitations that must be acknowledged. First, the system’s performance inherently depends on the quality and reliability of the sensory inputs. Sensor noise or malfunctions can affect the accuracy of the stimulus perception, which is not isolated from the BIAS itself. Additionally, the system does not eliminate false positives completely; while the IOR mechanism progressively downregulates long-duration activations, stimuli must persist long enough to be penalised appropriately. Therefore, short or spontaneous activations still influence the stimulus ranking and, as a consequence, produce non-expected expressions in the robot.

Another limitation concerns the assumption that stimulus intensity should decay over time. Although this behaviour mimics human attentional mechanisms, there might be situations where maintaining a constant attention to important stimuli over prolonged periods is desired. For example, when a temperature sensor indicates a risk of the robot overheating, additional conditions should be defined to maintain those stimuli without reducing their importance until any action has been taken to protect the robot. In such cases, it would be advisable to incorporate a safeguard mechanism that prevents critical signals from being deprioritised. In addition, as the third case study shows, sometimes the system repeats expressions (this occurred once in the experiment) if the stimulus importance does not decay fast enough and there is no other active stimulus. This issue can be corrected using thresholds that limit the activation of responses due to stimuli with shallow importance. However, raising the threshold value might also cause the robot to miss expressions when stimuli overlap.

From a computational perspective, the RL algorithm increases the computational needs with the number of stimuli, as all stimulus values are updated at each time step. Furthermore, the model relies on hyperparameters—such as the learning rate (α) and the sensitivity parameters of the IOR and AF functions ($w_{ior}$ and $w_{a\;f}$)—which have been empirically tuned for the current case study. Their optimal values may vary depending on the scenario or application and can be tuned using the procedure presented in this paper to obtain the desired response times.

Finally, although the system provides a perceptual and functional evaluation of attentional mechanisms, there is currently no standardised benchmark to validate the correctness of the resulting stimulus ranking objectively. This fact limits how the results can be generalised or compared to alternative implementations of attention systems.

## 8. Conclusions

This work presents a system that manages and prioritises sensory stimuli in real time within social robots engaged in embodied human–robot interaction. The BIAS system was developed in response to several perception challenges observed in such platforms: sensor false positives; overstimulation resulting from intense physical contact, which can simultaneously activate multiple sensors and lead to incoherent robot behaviours; and sensor saturation, which hinders the system’s ability to select contextually relevant stimuli at each moment of interaction.

This application-oriented work addresses these challenges by implementing two neurocognitive mechanisms—Inhibition of Return and Attentional Fatigue—which are formally encoded into the reward function of an existing RL algorithm. This definition allows the RL model to adjust the importance assigned to each stimulus dynamically, enabling the system to reduce redundant or persistent stimuli and detect novel and salient inputs more easily. The system accomplishes this capacity by dynamically ranking active stimuli and prioritising the most relevant one throughout the interaction.

The system has been successfully integrated into a pet robot companion, allowing it to react more adaptively and coherently to complex scenarios, such as receiving multiple caresses or being held in someone’s arms. Three representative case studies have been presented to validate the system’s performance, demonstrating its capacity to filter irrelevant inputs and select the most meaningful stimuli according to the current sensory context. From a human–robot interaction perspective, this approach enhances the perceptual robustness and contextual sensitivity of pet robots operating in naturalistic social environments. In addition, we compare the BIAS with a Baseline System to show how the robot’s behaviours significantly improve the number of expressions managed and the time they need to be executed, reducing delays that might affect the robot’s naturalness.

## 9. Future Work

Several directions can be explored further to improve the performance and applicability of the proposed system. First, it would be interesting to analyse the influence of the different parameters that play a role in the model, comparing the proposed model with other habituation computational methods available in the literature to provide deeper insights into its convergence properties and parameter sensitivities.

In line with human–robot interaction, conducting user studies would help analyse the system’s impact on perceived robot expressiveness and responsiveness, comparing scenarios with and without the bioinspired attention mechanism. This will provide insights into attentional modulation’s subjective and social implications in real-world human–robot interaction.

From a technical perspective, alternative Deep RL architectures will be investigated to overcome the computational cost of updating all stimuli at each time step. More scalable models with multi-input and multi-output capacities could allow for broader generalisation and faster adaptation. Additionally, future versions of the system may incorporate further biologically inspired processes, such as internal states reflecting the physical or cognitive fatigue of the robot, enabling attentional modulation based on both external and internal cues.

Finally, the system will be adapted and evaluated in other robotic platforms beyond the current pet robot companion, especially in scenarios involving continuous social contact and sensory saturation. Such deployments will validate the system’s versatility and robustness in diverse embodied interaction contexts.

## Figures and Tables

**Table 1:** Comparison of computational approaches for modelling attention and stimulus importance.

Algorithm	Attention	Bioinspired	Prediction Error	Novelty or Persistence
Classical Habituation	Partially	Partially	No	Yes (via repeated stimulation)
Bayesian Methods	Yes	Partially	No	Yes (via new evidence)
Rescorla–Wagner	No	Yes	Yes	No
Mackintosh	Yes	Yes	No	No
Pearce–Hall	Yes	Yes	Yes	Yes (via prediction error)
Bayesian Surprise	Yes	Partially	No	Yes (via new information)
Our approach	Yes	Yes	Yes	Yes (via fatigue and inhibition)

**Table 2:** Quantitative results for α tests. The bolded row indicates the final α selected for the model.

α	Fall Duration (s)	Fall Slope (Importance/s)
0.1	20.16	0.0496
0.3	19.30	0.0518
0.5	19.16	0.0522
0.7	19.05	0.0525
0.9	18.99	0.0526

**Table 3:** Quantitative results for $w_{ior}$ tests. The bolded row indicates the final $w_{ior}$ selected for the model.

$w_{\mathrm{ior}}$	Fall Duration (s)	Fall Slope (Importance/s)
0.005	1.705	0.586
0.015	2.150	0.465
0.05	5.203	0.192
0.1	10.148	0.099
0.5	>15	<0.066

**Table 4:** Quantitative results for $w_{a\;f}$ tests. The bolded row indicates the final $w_{a\;f}$ selected for the model.

$w_{a\;f}$	Fall Duration (s)	Fall Slope (Importance/s)
10	12.92	0.077
20	19.79	0.051
30	29.65	0.034
40	39.32	0.025

**Table 5:** Stimuli, related gestures, and time consumption for robot expressions. The arrows represent the positions that the motors take to perform a movement and the transitions between them.

Stimulus	Gesture Name	Actuation	Time
ASR: “Hello”	Wake up	- Sound: Snore - LEDs: Pink - Vibration: off - Motor right ear: 1 → 0 - Motor left ear: −1 → 0 - Motor tail: 0.5 → −0.5 → 0	6 s
ASR: “Go to sleep”	Sleep	- Sound: Snore - LEDs: White - Vibration: off - Motor right ear: 0.5 → 0 - Motor left ear: 0.5 → 0 - Motor tail: 0.5 → 0	4.5 s
RFID: “Toy”	Play	- Sound: Whistle - Vibration: off - Motor right ear: −1 → 0 - Motor left ear: 1 → 0	3 s
IMU	Movement	- Sound: Purring - LEDs: Yellow - Vibration: on - Motor tail: 0.5 → −0.5 → 0	4.5 s
Right ear	Touch right ear	- Sound: Purring - LEDs: Orange - Vibration: on - Motor right ear: 1 → −1 → 0	3 s
Left ear	Touch left ear	- Sound: Purring - LEDs: Orange - Vibration: on - Motor left ear: −1 → 1 → 0	3 s
Head	Touch head	- Sound: Purring - LEDs: Orange - Vibration: on - Motor right ear: −1 → 0 - Motor left ear: 1 → 0	3 s
Back	Touch back	- Sound: Purring - LEDs: Orange - Vibration: on - Motor right ear: 1 → 0 - Motor left ear: −1 → 0	3 s
Whiskers	Touch whiskers	- Sound: Purring - LEDs: Orange - Vibration: on - Motor tail: 0.5 → −0.5 → 0	2 s

**Table 6:** Metrics obtained in the third scenario with and without BIAS.

Metric	Baseline System	BIAS
Total expressions	85	9
Expressions in the queue	μ=38.29, σ=24.67	μ=1.21, σ=1.63
Time to completion	μ=128.31, σ=72.48	μ=8.31, σ=3.80
Computational consumption	μ=1.38, σ=1.05	μ=6.98, σ=2.38

## Data Availability

The original contributions presented in this study are included in the article. Further inquiries can be directed to the corresponding author.
